# Trends and Advances in Electrochemiluminescence Nanobiosensors

**DOI:** 10.3390/s18010166

**Published:** 2018-01-09

**Authors:** Mohammad Rizwan, Noor Faizah Mohd-Naim, Minhaz Uddin Ahmed

**Affiliations:** 1 Biosensors and Biotechnology Laboratory, Chemical Science Programme, Faculty of Science, Universiti Brunei Darussalam, Jalan Tungku Link, Gadong BE 1410, Brunei Darussalam; 14h0651@ubd.edu.bn (M.R.); faizah.naim@ubd.edu.bn (N.F.M.-N.); 2 Institute of Health Sciences, Universiti Brunei Darussalam, Jalan Tungku Link, Gadong BE 1410, Brunei Darussalam

**Keywords:** electrochemiluminescence, label-free, nanostructure materials, nanomaterials, nanocomposites, nanobiosensors, metal nanoclusters, ordered mesoporous carbon, signal amplification

## Abstract

The rapid and increasing use of the nanomaterials (NMs), nanostructured materials (NSMs), metal nanoclusters (MNCs) or nanocomposites (NCs) in the development of electrochemiluminescence (ECL) nanobiosensors is a significant area of study for its massive potential in the practical application of nanobiosensor fabrication. Recently, NMs or NSMs (such as AuNPs, AgNPs, Fe_3_O_4_, CdS QDs, OMCs, graphene, CNTs and fullerenes) or MNCs (such as Au, Ag, and Pt) or NCs of both metallic and non-metallic origin are being employed for various purposes in the construction of biosensors. In this review, we have selected recently published articles (from 2014–2017) on the current development and prospects of label-free or direct ECL nanobiosensors that incorporate NCs, NMs, NSMs or MNCs.

## 1. Introduction

### 1.1. Biosensors

A biosensor is a powerful and innovative analytical tool, which integrates a biological recognition element with a physio-chemical transducer to detect biological substances. The International Union of Pure and Applied Chemistry (IUPAC) defines a biosensor as “a device that uses biochemical reactions mediated by isolated enzymes, organelles or whole cells to detect the effects of chemical compounds by electrical, thermal or optical signals” [1,2]. The world’s first biosensor was invented in 1962 by Clark and Lyons to electrochemically measure glucose in biological samples [3]. The sensor utilized the glucose oxidase enzyme as a recognition element in order to detect reduction in oxygen levels or production of hydrogen peroxide. These levels would act as a read-out for glucose level in the body due to their correlation to each other [4,5]. 

**Figure 1:**
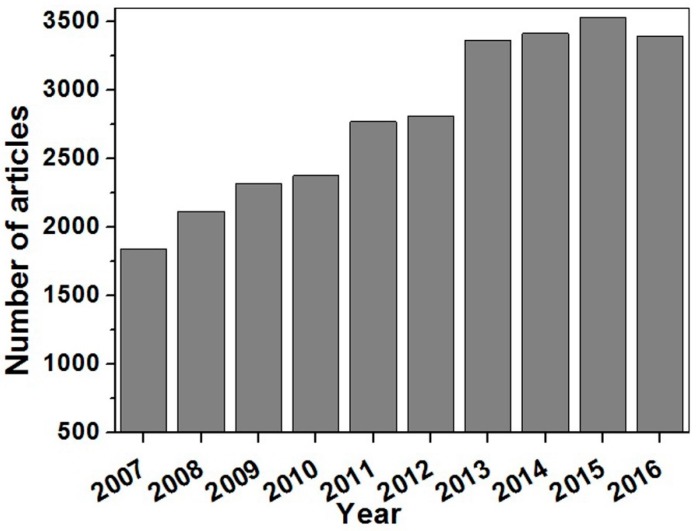
Number of articles published on ECL biosensors in the last 10 years with the keyword search of “electrochemiluminescence biosensor”, either in the title or in the abstract [26].

Since, their discovery Clark and Lyons, biosensors have gained a prominence that is evident by their extensive use in various fields. Figure 1 specifically shows the number of research articles that have been published on electrochemiluminescence (ECL) biosensors in the last 10 years. Some of the important applications of biosensors include medical diagnosis [6,7,8,9,10,11], the food industry [12,13,14,15,16,17], biomedicine [18], forensic science [19,20,21] environmental monitoring [16,22], water characteristic testing [23], defense and the military [24], as well as security [25]. In real-world settings, biosensors have replaced many tedious, expensive and complex conventional analytical techniques. Perhaps another reason for their popularity amongst researchers across the globe is that they create a way to unite different fields of biology, chemistry, physics, material sciences, nanosciences, electronics and optics [6,7,19] to allow for a multidisciplinary cooperation. 

### 1.2. Biosensor Components 

A biosensor is a device that consists of two fundamental components: a bioreceptor and a transducer [2]. A bioreceptor is a biological factor that recognizes and consolidates biomolecules or the target analytes that is in intimate contact with the transducer [27]. Bioreceptors may contain nucleic acids (DNA or RNA), antibodies, enzymes, cell receptors, organelles, synthetic receptors, microorganism, tissues, and organs [28,29]. 

**Figure 2:**
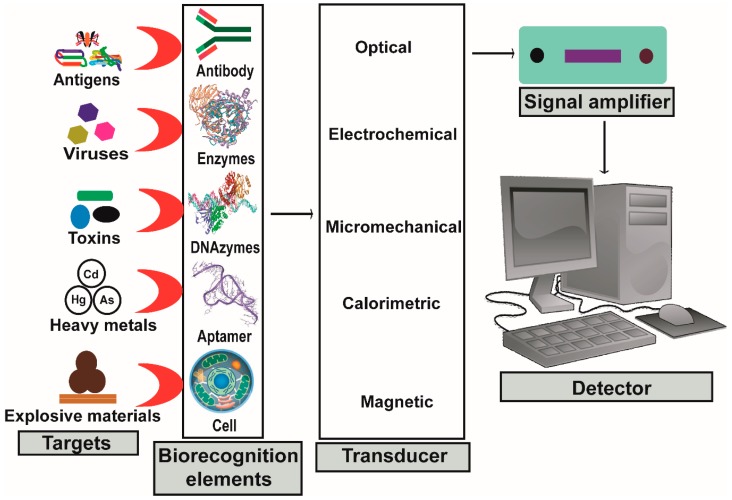
Illustrated scheme of a biosensor and its fundamental components.

The other important component of biosensor is a transducer, a physicochemical detector that measures the physical change that occurs between the analyte and the bioreceptor and then transforms that energy into a measurable electrical signal equivalent to a single analyte. The transducer is connected to the detector system through a connector whereby signals become amplified, analyzed and converted to concentration units. These data are then displayed by the device or stored within the device for later extraction [30]. All fundamental components of the biosensor are illustrated in Figure 2.

### 1.3. Construction of Biosensors

#### 1.3.1. Immobilization of Biomolecules

**Figure 3:**
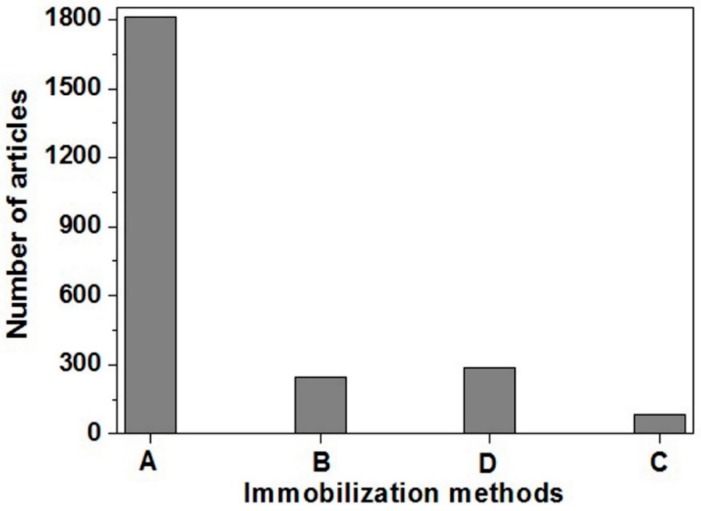
Research articles published in the last 10 years with the following keyword search: (A) “biosensor using adsorption,” (B) “biosensor using encapsulation,” (C) “biosensor using crosslinking” and (D) “biosensor using covalent bonding”, either in the title or in the abstract [26].

**Figure 4:**
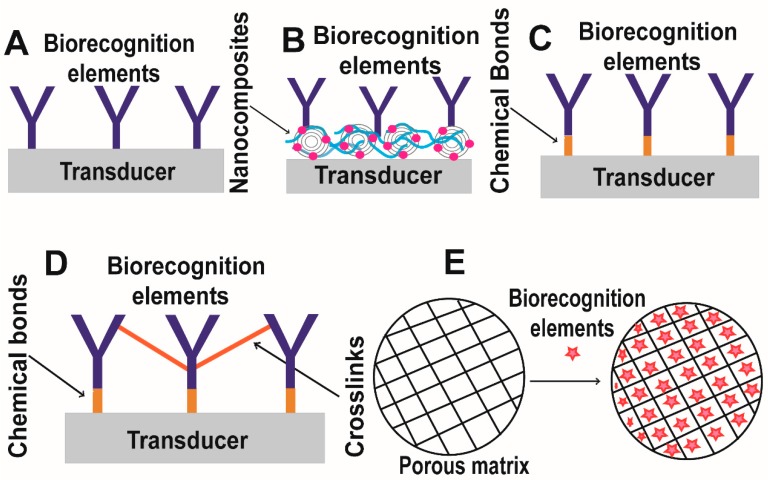
Illustrated scheme of event when physically adsorbing biomolecules on the surface of (**A**) transducer; (**B**) nanocomposites-modified transducer; (**C**) covalently attaching biorecognition elements on the transducer; (**D**) crosslinking between biorecognition elements and the transducer; and (**E**) biorecognition elements entrapment in polymer matrix.

The first and foremost step in the construction of a biosensor is the adherence of biomolecules onto the transducer, a step referred to as immobilization. The two most common methods of immobilization are physical methods (such as adsorption and encapsulation) [6,7,31,32,33] and chemical methods (such as crosslinking and covalent bonding) [34,35,36,37]. Each technique has its own advantages and limitations. The most frequently used technique for immobilization of bioreceptor is adsorption. Figure 3 illustrates the number of articles which utilized adsorption techniques in comparison to encapsulation, cross-linking and covalent bonding [6,7,31,38]. Therefore, in this review we have focused on the adsorption-based ECL nanobiosensors. The adsorption method of immobilization is a simple and straightforward technique. The process is quick, without requiring any complex reactions. Moreover, no hazardous chemicals are used, ensuring that the functionality of biomolecules is not changed [6,7,31]. An illustration depicting immobilized biomolecules on transducer “without nanocomposite modification” and “with nanocomposites modification” via adsorption are shown in Figure 4A,B, respectively. Covalent bonding and cross-linking techniques via chemical immobilization method of biomolecules are respectively shown in Figure 4C,D. During encapsulation, biomolecules are first enclosed in a porous polymer matrix followed by immobilization on the transducer surface, as shown in Figure 4E. 

#### 1.3.2. Transducer

The selection of the suitable biomolecule immobilization method is usually followed by deciding on the transducer for an effective bio/immunosensor. A transducer is a component which converts chemical [6,31], optical [7,15], mass [39] or temperature [40] signal into an electrical signal (see Table 1). Henceforth, depending on the types of transducer or the signal transduced, biosensors are mainly divided into electrochemical, optical, thermal, and piezoelectric devices. Essentially, an electrochemical biosensor generates an electrical signal when analytes interact with biomolecules to produce chemical changes on the chemically active area of the electrodes in the presence of an electrochemical probe. An optical biosensor, on the other hand, sense the change in the properties of electromagnetic wave when analytes communicate with biomolecules. Some examples of optical biosensors include those that are: surface plasmon resonance-based, fluorescence-based, chemiluminescence-based, and ECL-based. Thermal biosensors convert temperature change into electrical signal when analytes and biomolecules interact, while piezoelectric biosensors detect the difference in mass due to formation of a complex between analytes and biomolecules. 

It is clear that the immobilization of biomolecules and the accompanying transduction method are the fundamental components of biosensor fabrication. Hence, researchers are continuously introducing new combinations of biomolecule immobilization methods coupled with quick and sensitive transduction methods to fabricate biosensors with advanced analytical properties. Some important analytical parameters of biosensors that are progressively being improved include limit of detection, speed of response, signal amplification, sensitivity and specificity, detection range, selectivity, resistance to interference, stability, capacity for regeneration and cost-effectiveness. Henceforth, this review will cover the more recent research articles (from 2014–2017) whereby nanostructure materials (NSMs)- or nanomaterials (NMs)- or metal nanoclusters (MNCs)-based nanocomposites (NCs) have been utilized in the fabrication of ECL nanobiosensors.

## 2. Electrochemiluminescence

**Figure 5:**
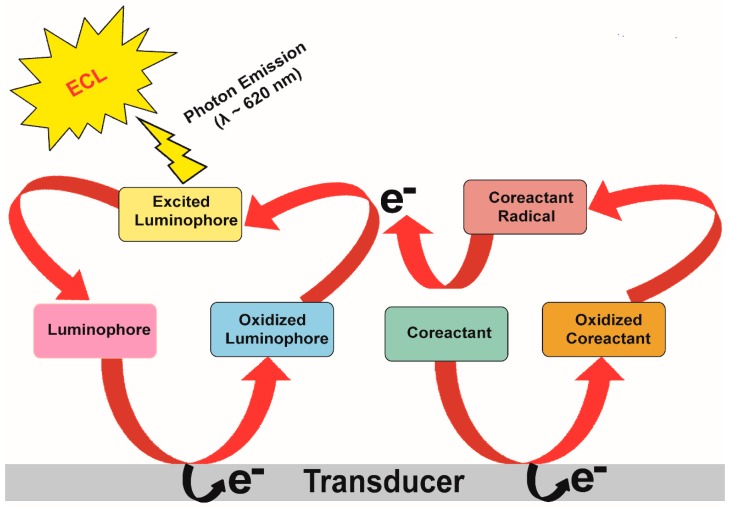
Schematic illustration of ECL mechanism and its generation on electrode surface in presence of suitable luminophore and co-reactant.

Electrochemiluminescence (ECL), alternatively known as electrogenerated chemiluminescence, is a technique whereby chemiluminescence is emitted upon electron-transfer reaction as depicted in Figure 5. 

ECL is highly versatile due to the ability to adjust these different parameters during the fabrication steps: potentials, currents, reaction time and electrode size. In terms of analytical ECL studies, co-reactant ECL predominates over its counterpart, annihilation ECL, due to the ease of ECL generation in a simple and environmentally safe aqueous solutions through a co-reactant pathway, as well as a wide range of ECL co-reactants to choose from. The method is, therefore, transferable to a wide range of assays for different purposes. ECL has diverse analytical applications, such as identifying co-reactants, ECL enhancers and ECL quenchers, or used in biomolecules detection, food and drug analysis, clinical diagnosis and environmental monitoring due to its high sensitivity, broad linear range and rapid analysis. ECL has also been coupled to other analytical techniques, such as flow injection analysis, high-performance liquid chromatography (HPLC), capillary electrophoresis, and micro total analysis (mTAS) [7,8,15,41,42,43]. The widespread use of ECL biosensors for biomolecule detection in various fields in the last decade has seen numerous adaptations incorporated in the fabrication of ECL biosensors, specifically to modify electrodes, ECL labels and ECL-emitting probes with different NSMs or NMs or MNCs and their composites. Although numerous review articles have been published on ECL and ECL biosensors [44,45,46,47,48,49,50,51,52,53,54], no recent review article includes the latest development on NSMs-, NMs- MNCs- and NCs-based ECL biosensors. Therefore, this review will specifically emphasize on the advancement in direct or label-free NSMs- or NMs- or MNCs nanocomposites-based ECL nanobiosensors from the year 2014 to 2017. NSMs- or NMs or MNCs-based nanocomposites are used for the fabrication of ECL immunosensors to enhance the following specifications: adsorption of recognition element on electrode, limit of detection, speed of response, signal amplification, sensitivity, detection range, selectivity, interference resistance, specificity, stability and cost-effectiveness. The main approach involves increasing the surface area and electron conductivity of the electrode surface. The selection of research articles cited in this review is arbitrary due to the large number of excellent and original research papers that have been published on this subject matter.

## 3. Mechanism of ECL Detection

ECL detection occurs by means of two well-known pathways [45,47,50,51,52,53,54]: (a) ion annihilation pathway and (b) co-reactant pathway.

(a) Ion annihilation pathway

Ion annihilation involves the generation of ECL by the formation of electrochemically generated and sufficiently stable intermediate cationic (R**˙**^+^) as well as anionic (R**˙**^−^) species by the emitter (R) at the electrode surface. These two oppositely charged radical ions annihilate one another to produce the excited state (R*) and the ground state (R). The excited state (R*) will emit light of wavelength *hv* to produce ECL and come to the ground state (R) as described in Scheme 1.

(b) Co-reactant pathway

The co-reactant pathway of ECL generation involves the presence of two species: emitter (R) and the co-reactant (C). Co-reactant ECL is generated by applying an anodic or cathodic potential in a solution containing the luminophore and the co-reactant. Therefore, both luminophore and co-reactant species can be oxidized or reduced at the electrode surface to form radical ions and intermediates species by changing the polarity of the applied potential. Finally, radical ions and intermediate species combined to form the excited state species, which emit light and can be detected by the ECL detector, such as a photomultiplier tube. Depending on the polarity of the applied potential, either highly reducing intermediate species are generated after electrochemical oxidation of co-reactant rendering the reaction as “oxidative-reduction”, or highly oxidizing intermediates are produced after electrochemical reduction rendering the reaction as “reductive-oxidation. The two possible mechanisms of the co-reactant ECL pathways are elucidated in Scheme 2 and Scheme 3 for (i) oxidative-reduction co-reactant ECL and (ii) reductive-oxidation co-reactant ECL, respectively.

(i) Oxidative-reduction co-reactant ECL pathway
(ii) Reductive-oxidation co-reactant ECL pathway

## 4. Nanostructure Materials, Nanomaterials, Metal Nanoclusters and Nanocomposite(s)

The current decade has seen tremendous development in nanosciences and nanotechnology in all areas of science and technology. Nanoscience or nanotechnology has gained particular importance in areas such as biomedical sciences [55], food industry [56], environment [57], catalysis [58], optics [59], and energy science [60]. To a large extent, this implicated the field of analytical chemistry, specifically in the fabrication of ECL nanobiosensors. The direct or indirect application of NSMs or NMs or MNCs as NCs mainly affect the limit of detection, sensitivity, range, selectivity, specificity and stability. Additionally, the use of NMs, NSMs and MNCs in NCs consequently results in the possibility of miniaturizing the devices with reduced volume of recognition element and sample needed, coupled with improved efficiency. NMs can enhance biosensor sensitivity by either enhancing the conductivity of the sensor platform or providing increased surface area for antigens or recognition elements due to their unique physical and chemical properties [7]. 

**Figure 6:**
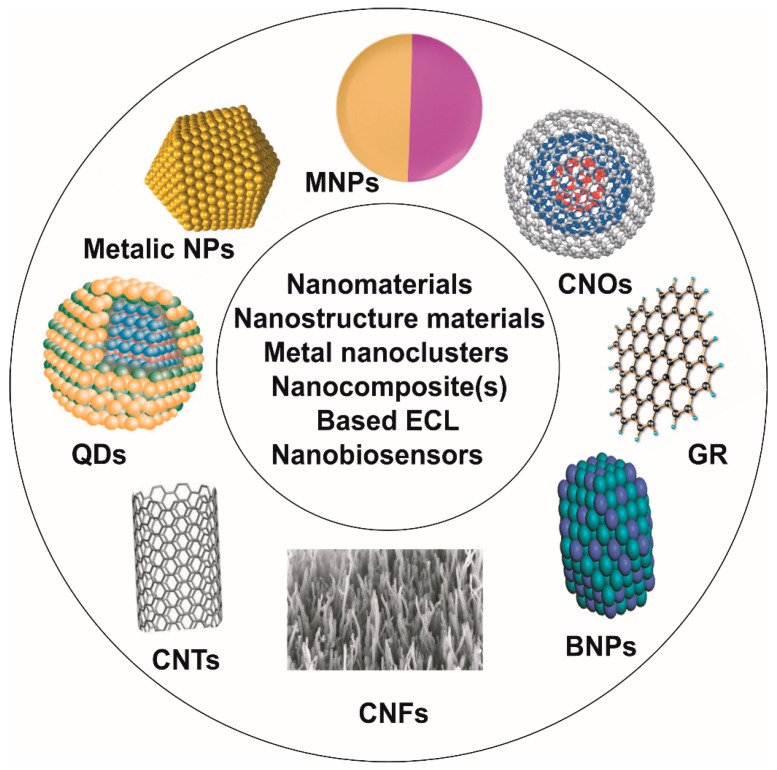
Illustration of various nanostructure materials or nanomaterials or metal nanocluster or nanocomposite being used for the fabrication of ECL nanobiosensors (MNPs: Magnetic nanoparticles, CNOs: Carbon nano-onions, GR: Graphene, BNPs: Bimetallic nanoparticles, CNFs: Carbon nanofiber, CNTs: Carbon nanotubes, QDs: Quantum dots, NPs: Nanoparticles).

Figure 6 demonstrates the application of the NSMs or NMs or MNCs as nanocomposites being recently used in the fabrication of ECL nanobiosensors. The use of NSMs or NMs or MNCs have been previously reviewed in the context of ECL-based nanobiosensors fabrication [44,48,52,53,54]. However, in this particular review article, we have selected some of the more recent articles (from 2014 to 2017) that utilize metallic, magnetic (AuNPs, AgNPs and Fe_3_O_4_), quantum dots (CdS, CdSe, CdTe and graphene), carbon (ordered mesoporous carbons, graphene, carbon nano-onions, and carbon nanotubes) NSMs, NMs and MNCs (Au, Ag and Cu) composites in the fabrication of ECL nanobiosensors for point-of-care devices for diagnostic applications.

## 5. Metal- and Magnetic Nanocomposite-Based ECL Nanobiosensors

Due to the unique property of noble metals (gold and silver) and magnetic nanomaterials, they have been used as nanocomposites in the fabrication of ECL nanobiosensors [61,62,63]. Some of these unique physicochemical properties are increased surface area and mechanical strength, in addition to their substantial electronic and catalytic properties. These properties have been exploited in order to detect biomolecular recognition events through electrical signals or electrochemical transductions. This has driven the development of a new generation of electronic nanobiosensors devices, which rely on changes in the ohmic response of an electrical circuit or the flow of electrons arising from faradaic current generated by oxidation or reduction near the surface of an electrode in order to measure the presence or concentration of biological molecules [64]. Moreover, the large surface area and high mass transference of MNPs lead to their integration as transducer materials in the fabrication of nanobiosensors [65].

### 5.1. Gold Nanoparticles (AuNPs)

**Figure 7:**
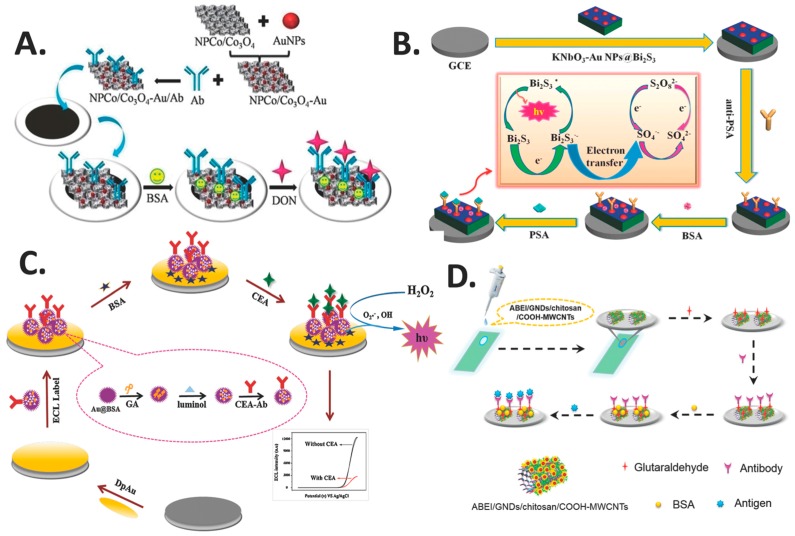
AuNPs nanocomposite-based ECL nanobiosensors: (**A**) ECL nanobiosensor with gold functionalized nonporous CO/Co_3_O_4_. Reprinted from Ref. [14] with permission from Elsevier; (**B**) ECL nanobiosensor incorporating the nanocomposite of KNbO_3_-AuNPs@Bi_2_S_3_. Reprinted from Ref. [70] with permission from Elsevier; (**C**) ECL nanobiosensor utilizing the nanocomposite of Au@BSA. Reprinted from Ref. [67] with permission from Elsevier; and (**D**) ECL nanobiosensor with ABEI/GNDs/chitosan/COOH-MWCNTs. Reprinted from [71] with permission from the American Chemical Society.

AuNPs is one of the most extensively studied NMs or NSMs in nanoscience and nanotechnology when compared to other metal-based nanomaterials. Monumental research and advanced applications of AuNPs have emerged only in the recent decade. Its highly favorable properties, which include a large surface area-to-volume ratio, unique optical and electronic properties, and easy surface modification, have contributed to the intensive focus on AuNPs from both academia and industry. Much effort has been devoted to tailoring the properties of AuNPs for specific biosensor applications [66]. AuNPs have been employed in ECL nanobiosensors as nanocomposites due their unique biocompatibility [67], high electrical conductivity, size-related electronic, magnetic and optical properties, catalysis and the ease by which they can self-assemble [68]. The unique properties of AuNPs-modified electrode interfaces lead to the development of novel ECL nanobiosensors with high sensitivity and good stability [7], which is mainly attributed to the increased surface area of the electrodes [69]. Table 2 summarizes the AuNPs-based NCs recently used in the fabrication of ECL-based direct or label-free nanobiosensors. Lv et al. fabricated an ECL nanobiosensor (Figure 7A) using AuNPs functionalized nonporous Co/Co_3_O_4_ (NPCo/Co_3_O_4_-Au) NCs to modify the electrodes for detecting mycotoxin deoxynivalenol (DON). NCs of NPCo/Co_3_O_4_ and AuNPs enhanced the conductivity of the electrodes and their catalytic properties for the luminescence of RuSi@Ru(bpy)_3_^2+^, resulting in a highly sensitive ECL nanobiosensor. The ECL electrodes exhibited a wide linear range of activity for DON from 5 pg mL^−1^ to 100 ng mL^−1^, with the ability to detect very low concentration of DON at 1 pg mL^−1^ and displaying excellent stability and reproducibility [14]. In another study, Li et al. used AuNPs to prepare the NCs niobate-Au nanoparticles@bismuth sulphide (KNbO_3_-AuNPs@Bi_2_S_3_) to modify glassy carbon electrode (GCE) for the detection of prostate specific antigen (PSA) (Figure 7B). AuNPs were combined with Bi_2_S_3_ via the Au-S covalent bond and anti-PSA via Au-NH_2_ covalent bond without crosslinking. AuNPs elevated the electron transfer efficiency of the nanocomposite at the surface of the electrode, which in turn enhanced the sensitivity and improved both the ECL signal produced and the stability of the ECL nanobiosensor. The study reported that the ECL nanobiosensor exhibited high sensitivity, selectivity, good repeatability and long-term stability, with a wide detection range of 0.005 to 5 ng mL^−1^ and a low detection limit of 3 pg mL^−1^ [70]. 

In another report by Zhang et al., a novel ECL nanobiosensor that utilized multifunctionalized flower-like Au@BSA nanoparticles was created for the determination of tumor marker carcinoembryonic antigen (CEA). The Au@BSA nanoparticles provided a large surface area, good electrical conductivity and excellent biocompatibility as an ECL interface. Moreover, they are also ideal carriers for the immobilization of luminol molecules that act as signal probes, and CEA capture antibodies that act as molecule recognition probes (Figure 7C). The nanobiosensor was reported to achieve good stability, excellent reproducibility and favorable selectivity. A wide detection range of 0.001 to 200 ng mL^−1^ was exhibited by the nanobiosensor, with a low limit of detection of 0.0003 ng mL^−1^ [67]. In a separate report, another group fabricated an ECL nanobiosensor using N-(aminobutyl)-N-(ethylisoluminol) (ABEI)-functionalized gold nanodots/chitosan/multiwalled carbon nanotubes (ABEI/GNDs/chitosan/COOH-MWCNTs) nanocomposite-modified ITO electrodes. Grafting the nanocomposite onto the electrode was possible due to the film-forming property of the nanocomposite. This is followed by the conjugation of N-Terminal Pro-brain natriuretic peptide antibody (anti-NT-proBNP) onto the modified surface using glutaraldehyde (Figure 7D). Strong and stable ECL signal was produced by the nanobiosensor with a wide linear range of 0.01–100 pg mL^−1^ and a limit of detection of 3.86 fg mL^−1^, which is three orders lower than the electrochemistry method reported previously. The ECL nanobiosensor also demonstrated high sensitivity, high selectivity coupled to simple and fast analysis [71].

### 5.2. Silver Nanoparticles (AgNPs)

**Figure 8:**
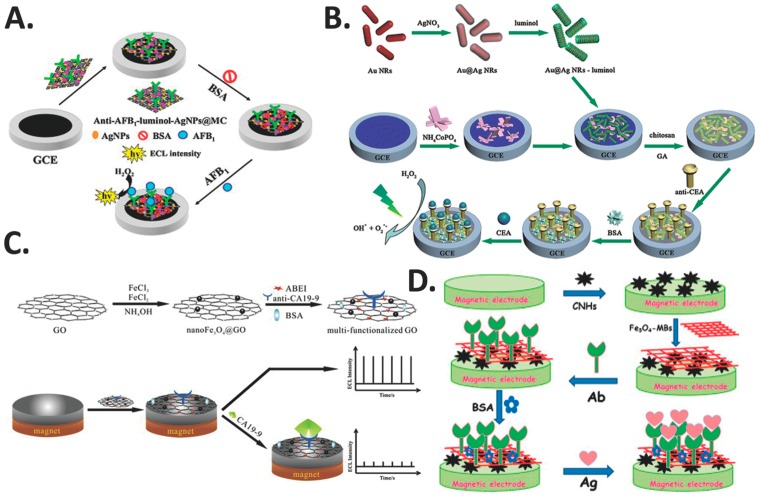
AgNPs nanocomposite-based ECL nanobiosensors: (**A**) ECL nanobiosensor with nanocomposite of luminol-AgNPs@OMC. Reprinted from Ref. [62] with permission from Elsevier; (**B**) ECL nanobiosensor with nanocomposite of NH_4_CoPO_4_/Au@Ag-luminol. Reprinted from Ref. [78] with permission from Springer; (**C**) ECL nanobiosensor with nanocomposite of nanoFe_3_O_4_@GO. Reprinted from Ref. [79] with permission from Elsevier; (**D**) ECL nanobiosensor with nanocomposite of Fe_3_O_4_-NFs. Reprinted from [81] with permission from Elsevier.

Among the noble metal nanomaterials, AgNPs are capable of facilitating electron transfer from the reaction center to the electrode surface, which makes them capable of higher intensity electron transfer. AgNPs have few desirable properties such as their ease-of-functionalization, good biocompatibility, ease of immobilization of biomolecules and its ability to increase ECL intensity of luminophore [62]. These facts have led AgNPs to be incorporated in ECL nanobiosensor fabrication. Some of the currently fabricated ECL nanobiosensors using AgNPs are summarized in Table 2. Lv et al. used luminol functionalized with silver nanoparticles loaded onto ordered mesoporous carbon (luminol-AgNPs@OMC) electrode to conjugate AFB1 antibody (anti-AFB1) onto the sensing platform for the ECL-based detection of the AFB1 (Figure 8A). The AgNPs were used to improve ECL signal, absorption capacity of antibody, and catalysis of the luminescence of luminol. The ECL nanobiosensor demonstrated fast, sensitive, specific, stable and reliable results for the detection of AFB1 with a linear range of 0.1 pg mL^−1^ to 50 ng mL^−1^ and a low detection limit of 50 fg mL^−1^. Luminol-AgNPs@OMC proved to be an excellent sensing platform for the fabrication of simple and sensitive nanobioensors [62]. Zhang et al. constructed a label-free ECL nanobiosensor for the detection of CEA by employing NH_4_CoPO_4_/Au@Ag-luminol/chitosan matrix as antibody carriers. NH_4_CoPO_4_ was utilized for stabilizing the ECL system, while the Au@Ag nanorods act as a mimic enzyme for the catalysis within the luminol-H_2_O_2_ ECL system. The ECL nanobiosensor was fabricated by placing the materials down onto a GCE in the following order: chitosan film containing NH_4_CoPO_4_, Au@Ag and luminol, and immobilization of anti-CEA antibody (Figure 8B). In the presence of H_2_O_2_, ECL signal is generated when electrochemical reaction of luminol occurred on the surface of the Au@Ag-luminol film. The nanobiosensor was found to have a detection range of 0.1 pg mL^−1^ to 380 ng mL^−1^, with a low detection limit of 30 fg mL^−1^ [78].

### 5.3. Magnetic Nanoparticles (MNPs)

Currently, MNPs have become an emerging focus in the development and fabrication of sensors and biosensors. MNPs can be integrated into the transducer material or be dispersed in the sample. Due to its ability to be manipulated by magnetic fields, applying an external magnetic field will attract them onto the biosensor’s active detection surface, which can be best accomplished when MNPs are at sizes of 10–20 nm due to super-magnetism. Furthermore, MNPs possess large surface areas and high mass transference which enhance the sensitivity and stability in the fabrication of biosensors and other detection systems in clinical, food and environmental applications. Some recent applications of the MNPs in the fabrication of ECL nanobiosensors are presented in Table 2.

MNPs made of Fe_3_O_4_ have the advantage of being easily separated and immobilized [65]. Sha et al. reported the construction of an effective ECL naobiosensor for the ultrasensitive determination of (CA19-9) that utilized multi-functionalized graphene oxide anti-CA19-9/ABEI-nanoFe_3_O_4_@GO (Figure 8C). The presence of nanoFe_3_O_4_@GO extended the outer Helmholtz plane (OHP), which enabled all N-(4-aminobutyl)-N-ethylisoluminol (ABEI) molecules to be immobilized onto GO and became electrochemically active. This improved the effective emission of ECL signals and the detection sensitivity due to increased conductivity. In this set-up, anti-CA19-9 antibodies acted as the recognition element for CA19-9 and ABEI acted as the electrochemiluminophore. The nanobiosensor could detect concentrations of CA19-9 in the range of 0.001 to 5 U mL^−1^ and a detection limit of 0.0005 U mL^−1^ with satisfactory specificity, stability, reproducibility and regeneration [79]. In a different study, Xu et al. fabricated an ultrasensitive label-free ECL nanobiosensor for the detection of AFB1 based on magnetic nanoarchitecture (Figure 8D). By using magnetic control technology, magnetic nanofibers (Fe_3_O_4_-NFs) could be immobilized tightly onto CNHs matrix with the ability to accommodate a large number of antibodies on the interface. This afforded the nanobiosensor a high sensitivity for AFB1, a wide detection linear range from 0.05 ng mL^−1^ to 200 ng mL^−1^ and a low detection limit of 0.02 ng mL^−1^. Moreover, the ECL nanobiosensor exhibited good reliability, fast detection and good reproducibility at a low cost [81].

### 5.4. Metal Nanoclusters (MNCs)

Recently, MNCs such as Au, Ag, Cu, Pt, Pd, and some alloys have become promising materials for ECL investigations and the construction of nanobiosensors due to their unique features, such as chemical inertness, high electrical conductivity, low toxicity, easy labeling and excellent biocompatibility as well as stability [82,84]. MNCs are clusters of few atoms to a maximum 100 atoms, and depending on the number of atoms that exist within the cluster, their sizes lies typically in the range of sub-nanometer (less than 2 nm) to several nanometers (small nanoparticles) [83]. This size of MNCs is comparable to the Fermi wavelength of electrons in metal, leading to the continuous density of states splitting into discrete energy levels, which provide MNCs with attractive characteristics, such as excellent optical, electrical, electrochemical, magnetic, and chemical properties, leading to their use in the fabrication of ECL based nanobiosensors [84]. Table 2 summarizes the MNCs-based NCs recently used in the fabrication of ECL-based nanobiosensors. In these new systems, MNCs participate in ECL reactions as luminophores, quenchers and catalysts. In-addition to that, MNCs have low toxicity, high stability, good water solubility, easy preparation and biocompatibility, which make MNCs promising materials for ECL investigation.

Report have shown that among the MNC composites, the composite of AuNCs endows them with distinct ECL behavior, rendering them popular in electroanalytical applications [84]. Due to the fascinating features of the AuNCs such as chemical inertness, low toxicity, easy labeling and excellent stability, Luo et al. constructed an ECL nanobiosensor based on Au nanoclusters/graphene (AuNCs/GR) hybrid for the sensitive detection of pentacholophenol (PCP) using S_2_O_8_^2−^ as co-reactant. AuNCs/GR hybrid based-ECL nanobiosensor demonstrated a wide linear range from 0.1 fM to 0.1 nM with a low detection limit of 10 fM. Additionally, the constructed ECL-nanobiosensor demonstrated easier, simpler and sensitive detection of PCP in real water samples [82]. Meanwhile, Nie et al. prepared luminol functional AuNCs (lum-AuNCs) for the fabrication of the nanobiosensor to detect alkaline phosphatase (ALP). Compared to individual luminol, AuNCs, and the mixture of luminol and AuNCs, the composite of lum-AuNCs had special ECL properties, which caused up to 100-folds enhancement in ECL intensity than the ECL intensity of the mixture. The fabricated nanobiosensor exhibited a dynamic detection range of ALP from 0.3 to 12.0 nM with a low detection limit of 0.1 nM. Furthermore, this nanobiosensor displayed high specificity, good stability and great potential in the detection of ALP in human serum samples [83].

## 6. Quantum Dots Nanocomposite-Based ECL Nanobiosensors

**Figure 9:**
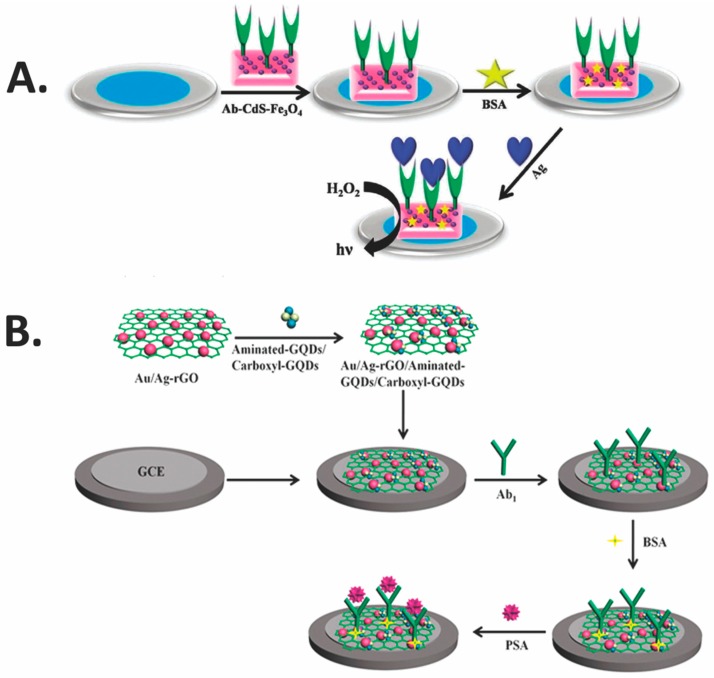
Quantum dots nanocomposite based ECL nanobiosensors: (**A**) ECL nanobiosensor with nanocomposite of CdS-Fe_3_O_4_. Reprinted from Ref. [80] with permission from the Royal Society of Chemistry; (**B**) ECL nanobiosensor with nanocomposite of Au/AgrGO. Reprinted from Ref. [38] with permission from Nature.

Quantum dots (QDs) are roughly spherical semiconductor nanoparticles or colloidal semiconductor nanocrystals that have unique optical, electronic and photophysical properties. They have been increasingly investigated in ECL mechanism study and has been applied in the labeling, imaging, and detection of biological material [85]. Some of the more extensively used QDs in ECL nanobiosensors are CdS, CdSe, CdTe and graphene quantum dots, as summarized in Table 3. Lv et al. designed an ECL nanobiosensor using the nanocomposite of CdS-Fe_3_O_4_ for the ultra-sensitive detection of ochratoxin A (OTA), as shown in Figure 9A. CdS QDs act as the ECL emitters and were immobilized onto mesoporous Fe_3_O_4_ along with anti-OTA antibodies. The ECL nanobiosensor exhibited high ECL signal intensity and sensitivity, with a detection limit of 2 pg mL^−1^ and a concentration range of 0.01 to 100 ng mL^−1^ [80]. In a separate study, Wu et al. constructed an ECL nanobiosensor for the detection of the PSA using nanocomposites of AuNPs, AgNPs and graphene quantum dots (Au/AgrGO) as sensing platform (Figure 9B). Aminated carboxyl Au/AgrGO was synthesized and loaded onto the electrode, which augmented the surface area for reaction and elevated electron conductivity in the presence of co-reactant K_2_S_2_O_8_. This was followed by the immobilization of anti-PSA through the adsorption of Au/Ag on proteins. The nanobiosensor demonstrated a detection range of 1 pg mL^−1^ to 10 ng mL^−1^, with a detection limit of 0.29 pg mL^−1^. The ECL nanobiosensor demonstrated superior stability, sensitivity, repeatability and selectivity [38].

## 7. Carbon Nanomaterials (CNMs)- or Carbon Nanostructured Materials (CNSMs)- Nanocomposite-Based ECL Nanobiosensors

Research in CNMs or CNSMs have advanced greatly in recent years, with a particular focus on graphene and carbon nanotubes (CNTs) for the development of carbon NCs-based biosensors. CNMs and CNSMs are attractive avenue as sensor components due to their exceptional electrical, thermal, chemical and mechanical properties, which render sensors more reliable, accurate and fast. Depending on the types of target molecules, different approaches can be taken in order to design a sensor device, making use of either zero-dimensional (carbon nano-onions) or one-dimensional (CNTs), or two-dimensional (graphene) or three-dimensional ordered mesoporous carbons (OMCs) or CNMs or CNSMs as NCs in order to fabricate a much improved analytical ECL-based nanobiosensors [89,90,91,92,93]. Presently, carbon nano-onions have emerged as the popular choice for the fabrication of sensitive and selective nanobiosensor due to their high surface area-to-volume ratio that provides a large surface area for biorecognition element binding, as well as improve electronic conductivity [90]. The latest examples of CNMs or CNSMs or OMCs NCs-based ECL nanobiosensors have been summarized in Table 4, with specific examples of each class of CNMs or CNSMs-based NCs.

### 7.1. Ordered Mesoporous Carbons (OMCs)

OMCs constitute a subclass of 3D nanostructured porous CNMs or CNSMs. These materials have recently attracted great attention of researchers in electroanalytical applications for the fabrication of highly sensitive ECL nanobiosensors due to their good electronic conductivity, chemical inertness, great porosity (high specific surface area, large pore volume and size) and widely open ordered structure made of uniform and tunable pore sizes, which ensure fast mass transport rates [62,93]. 

### 7.2. Graphene

**Figure 10:**
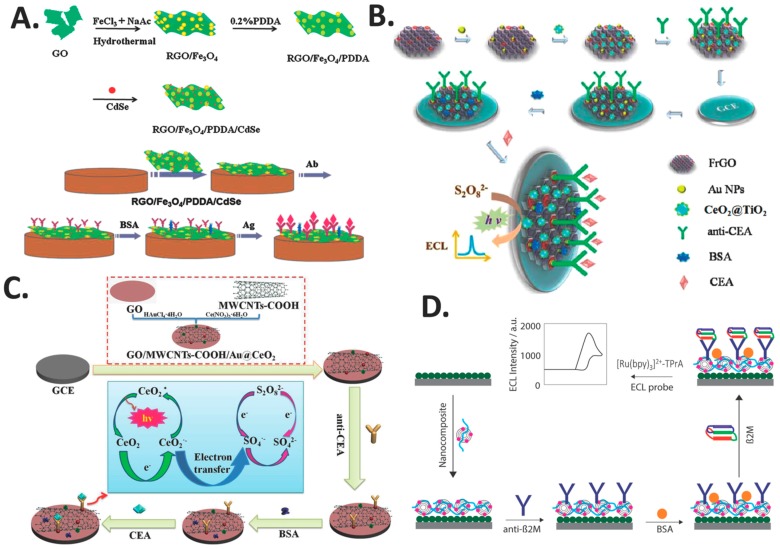
Carbon nanocomposite-based ECL nanobiosensors: (**A**) ECL nanobiosensor with nanocomposite of Au-FrGO and CeO_2_@TiO_2_. Reprinted from Ref. [10] with permission from Elsevier; (**B**) ECL nanobiosensor with nanocomposite of (RGO)/Fe_3_O_4_/PDDA/CdSe. Reprinted from Ref. [9] with permission from Elsevier; (**C**) ECL nanobiosensor with nanocomposite of GO/MWCNTs-COOH/Au@CeO_2_. Reprinted from Ref. [68] with permission from the American Chemical Society; (**D**) ECL nanobiosensor with nanocomposite of AuNPs@CNOs-CS. Reprinted from Ref. [7] with permission from the Royal Society of Chemistry.

Graphene, a two-dimensional (2D) carbon material of one-atom thickness, is one of the most exciting CNMs or CNSMs being researched today for its potential applications in developing a new generation of nanobiosensors due to its unique and novel properties. Its sp^2^ hybridization drives the carbon atoms in graphene to be in a closely packed, honeycomb lattice structure, acting as a fundamental building block for carbon-based nanomaterials such as the zero-dimensional (0D) CNOs, one-dimensional (1D) CNTs, and three-dimensional (3D) graphite. Graphene and its derivatives possess a few exceptional properties such as high thermal conductivity, superior tensile strength, tunable optical property, remarkable elasticity, and perhaps most importantly, is their exceptional electrical conductivity due to factors such as its high quantum Hall effect and electron-hole symmetry [89,96]. Yang et al. constructed a reduced graphene oxide (RGO)/Fe_3_O_4_/PDDA/CdSe nanocomposite-based ECL nanobiosensor (Figure 10A) for the detection of human IL-6. The two-dimensional RGO can be modified to improve electrical performance, and overall the nanobiosensor exhibited good magnetism, ECL properties and biocompatibility, and low toxicity. Additionally, high analytical performance is demonstrated by the nanobiosensor in terms of linear range, stability, reproducibility, selectivity and sensitivity. The ECL intensity demonstrated by the nanobiosensor decreased linearly with IL-6 concentrations in the range of 0.002–20 ng mL^−1^, with a limit of detection of 0.65 pg mL^−1^ [10]. In another report, Yang et al. constructed a composite to be incorporated into an ECL nanobiosensor that consisted of macroporous gold nanoparticles (AuNPs) functionalized reduced graphene oxide (rGO) capped Fe_3_O_4_ (Au-FrGO) and CeO_2_@TiO_2_ composite (Figure 10B) for the label-free detection of CEA. The microporous structure of FrGO was assembled by layering rGO sheets capped cationic Fe_3_O_4_ nanoparticles, culminating in a larger surface area for reaction compared to rGO. On its own, Fe_3_O_4_ maintained a rather low magnetism, but when combined with AuNPs and FrGO, it imparted the nanocomposite with a more favorable biocompatibility and electrical conductivity, favoring further immobilization of CeO_2_@TiO_2_ and antibodies. The capacity of applying magnetic separation further simplified the preparation procedure. The ECL nanobiosensor exhibited excellent stability, repeatability and selectivity, with a linear range of 0.01 pg mL^−1^ to 10 ng mL^−1^ and a limit of detection of 3.288 fg mL^−1^ [9].

### 7.3. Carbon Nanotubes and Carbon Nano-Onions

Since the significant discovery of carbon nanotubes (CNTs) in 1991, a flurry of research has focused on this specific allotrope of carbon. CNTs are well-ordered, high aspect ratio one-dimensional (1D) CNMs or CNSMs that can be rolled into seamless cylinders due to the sp^2^-hybridized carbon atoms in the graphene sheet. CNTs have diameters measuring in the nanometre range with lengths in the microns range. Two classes of CNTs have been identified, which are single-walled carbon nanotubes (SWNTs) and multi-walled carbon nanotubes (MWNTs). SWNTs are composed of only one rolled-up graphene layer (diameter 0.4–2 nm), whereas MWNTs are composed of multiple nested graphene layers of increasing diameter arranged in concentric tubes (2–100 nm) [89,96,97]. Due to their unique electronic, chemical, and mechanical properties, CNTs have been found to be useful in a wide range of applications. They have since become one of the more popular carbon nanomaterials for use in ECL biosensors as their conducting properties can accelerate electron transfer for electrochemical and ECL-based sensors and they can act as conducting pathways between luminophores and electrode. In addition to their excellent chemical and thermal stability, enhanced electrochemical reactivity, it has been reported that CNTs can accumulate important biomolecules, alleviate surface fouling effects, and their increased surface area and porosity result in faster diffusion of co-reactants [98,99,100]. 

Carbon nano-onions (CNOs) or onion-like graphitic particles or onion-like carbons (OLCs), also known as multi-shell fullerenes, are zero-dimensional (0D) carbon nanomaterials structured as concentric layers closed carbon shells, resembling an onion. Depending on the synthetic protocol implemented, a diameter of CNOs between 60 and 300 nm can be obtained, with a distance of 0.34 nm between each layer. The unique structure and properties of CNOs render them effective in a large number of applications, including incorporation into a biosensor. Like carbon nanotubes, CNOs generally exhibit poor solubility in both aqueous and organic solvents, hence they are mainly used in a composite form in nanobiosensor construction. In addition to possessing a high surface area-to-volume ratio and biocompatibility, CNOs can act as a linking layer between biomolecules and the surface of the sensor to amplify signals generated by the nanobiosensor [7]. Pang et al. constructed a highly sensitive ECL nanobiosensor in order to detect CEA as shown in Figure 10C. Nanocomposites of graphene oxide/carboxylated multiwall carbon nanotubes/gold/cerium oxide nanoparticles (GO/MWCNTs-COOH/Au@CeO_2_) were used to modify glassy GCE. The nanocomposite acted as anti-CEA antibody carriers and sensing platforms within the nanobiosensor, resulting in superior conductivity and large surface area for reaction. The GO/MWCNTs-COOH/Au@CeO_2_ nanocomposite-modified platform showed excellent cathodic ECL performance and sensitive response to CEA, with a limit of detection of 0.02 ng mL^−1^ and a wide linear range of 0.05–100 ng mL^−1^ [74]. Rizwan et al. designed a label-free ECL nanobiosensor for the detection of β2M based on AuNPs@CNOs-CS nanocomposite-modified QDs-SPE (Figure 10D). This nanocomposite greatly improved the electronic transmission rate and enhanced the capture of photons emitted from the luminophore tris(bipyridine)ruthenium(II) chloride ([Ru(bpy)^3^]^2+^Cl). Moreover, the biocompatible surface of the electrode possessed an increased effective surface area for capturing and binding of a large number of anti-β2M. These properties resulted in an ECL nanobiosensor that demonstrated 3 × 10^6^ higher sensitivity when compared to existing nanobiosensor, good reproducibility, long-term stability and selectivity, with a linear detection range of 1 fg mL^−1^ to 100 ng mL^−1^ and a low detection limit of 1 fg mL^−1^ [7].

## 8. Conclusions and Prospects

Due to the increasing applications of ECL-based nanobiosensors in medical diagnostics, pharmaceuticals, food quality control, environmental safety and security, NMs, NSMs, MNCs and NCs are increasingly being incorporated into biosensors. These nanomaterials allow for miniaturization of biosensors, low production cost, ease of use, the possibility of mass production, use of small sample volumes and high analytical performance. In this review article, we have summarized various NMs, NSMs, MNCs and NCs that have recently been used in the fabrication, construction, design and development of direct or label-free ECL nanobiosensors. Special emphasis has been placed on nanocomposite-modified sensing platform that are used in the fabrication of ECL-based nanobiosensors for medical diagnosis. Applications of NMs, NSMs, MNCs and NCs lead to various advantageous properties such as increased surface area for capture and binding of a large number of antibodies, ECL signal amplification, increased sensitivity, increased long term stability and improved electronic transmission rate [73]. Furthermore, these nanocomposites exhibited good selectivity, excellent biocompatibility, specificity, reproducibility and selectivity, improved magnetism, cost-effective and potential for regeneration of ECL nanobiosensors [7,67,79]. Despite these favorable characteristics, incorporation of novel NMs, NSMs, MNCs or polymer-based nanocomposite in biosensors are still required in order to further improve the sensitivity and the long-term stability of ECL nanobiosensors [7]. Currently, only very few label-free ECL-based nanobiosensors exploited polymer-based nanocomposite (such as: polyethylene glycol, chitosan and nafion), whereas materials such as MNCs, buckyballs, OMCs, carbon nanofibers, CNOs or SWCNTs have not been heavily utilized in the preparation of nanocomposites to modify the sensing platform of ECL-based nanobiosensors, which could increase the ECL signal intensity [17,84,93]. Similarly, disposable screen-printed electrodes and paper-based electrodes have not been employed as frequently in the preparation of nanocomposites in ECL nanobiosensors for point-of-care devices. Development of specialized ECL nanobiosensors, including enzymatic ECL nanobiosensors, ECL nano-immunosensors and ECL aptasensors, has great potential for practical applications, especially in the field of clinical diagnostics for the purpose of personalized medical monitoring. We predict that in the future, the demand for ECL-based nanobiosensors will be high due to their potential in a diverse range of applications. We anticipate the elimination of lengthy laboratory assays on bodily fluids (urine, blood, saliva and tears) as ECL-based nanobiosensors became a more sensible alternative approach in diagnosing various pathologies and diseases. 

## Figures and Tables

**Scheme 1:** Ion annihilation pathways.

**Table:** 

R − e^−^	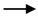	R**˙**^+^	(oxidation at the electrode surface)
R + e^−^	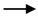	R**˙**^−^	(reduction at the electrode surface)
R**˙**^+^ + R**˙**^−^	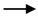	R + R*	(annihilation process/formation of excited state)
R*	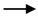	R + *hv*	(emission of light)

**Scheme 2:** Oxidative-reduction co-reactant ECL pathways.

**Table:** 

R − e^−^	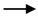	R**˙**^+^	(oxidation at the electrode surface)
C − e^−^	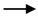	C**˙**^+^	(reduction at the electrode surface)
C + C	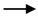	R + C**˙**	(homogenous chemical reactions)
C**˙**^+^	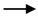	*C_r_	(homogenous chemical reactions)
*C_r_ + R	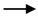	R**˙**^−^ + P	(homogenous chemical reactions)
R**˙**^−^ + R**˙**^+^	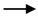	R + R*	Or R**˙**^+^ + *C_r_ 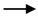 R* + P (excited state formation species)
R*	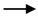	*hv*	(emission of light)

**Scheme 3:** Reductive-oxidation co-reactant ECL pathways. Here, *C_r_ and *C_o_ are the co-reactant intermediates for the reducing and oxidizing agents, respectively, and P is the product associated with *C_r_ and *C_o_ reactions, *h* is the Planck constant with a value of 6.634 × 10^−34^ J.s or 4.13 × 10^−15^ ev, *v* is the frequency (Hz) of the emitted light (*v* = c/λ, c = speed of light (3.00 × 10^8^ ms^−1^), and λ is wavelength of the emitted light).

**Table:** 

R + e^−^	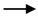	R**˙**^−^	(reduction at the electrode surface)
C + e^−^	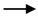	C**˙**^−^	(reduction at the electrode surface)
R**˙**^−^ + C	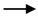	R + C**˙**^−^	(homogenous chemical reactions)
C**˙**^−^	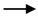	*C_o_	(homogenous chemical reactions)
*C_o_ + R	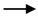	R**˙**^+^ + P	(homogenous chemical reactions)
R**˙**^−^ + R**˙**^+^	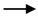	R + R*	Or R**˙**^+^ + *C_o_ 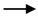 R* + P (excited state species formation)
R*	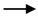	*hv*	(emission of light)

**Table 1:** Types of biosensor based on transducers/signals transduced.

Signal Type on Transducer	Signal Transduced	Type of Biosensors	Reference
**Chemical**	Electrical	Electrochemical	[6]
**Optical**	Electrical	Optical	[7]
**Mass change**	Electrical	Piezoelectric	[38]
**Temperature change**	Electrical	Thermal	[39]

**Table 2:** Metals- and magnetic-NMs- or NSMs- or MNCs- or NCs-based ECL nanobiosensors.

Electrode	NMs/NSMs	NCs	Function	Assay type	Bioanalyte	LOD	Range	Ref.
GCE	AuNPs	MWCNTs-Cys-AuNPs	Signal amplification, reproducibility, high sensitivity, stability and cost-effective	Direct	Diphen-hydramine hydrochloride (DPH)	6.7 × 10^−9^ M	2 × 10^−8^–7.5 × 10^−4^ M	[72]
QDs-SPE	AuNPs	AuNPs@CNOs/Chitosan	High ECL, increased effective surface area, high anti-β2M binding, enhanced photons capture, high detection limit, wide linear range, long-term stability and good selectivity	Label-free	Beta-2 microglobulin (β2M)	1 fg mL^−1^	1 fg mL^−1^–100 ng mL^−1^	[7]
GCE	AuNPs	MWCNTs-Au	Signal amplification, sensitivity, selectivity, reproducibility, stability and low cost	Direct	Bisphenol A	0.083 μM	0.25–100 μM	[61]
GCE	AuNPs	Au@BSA nanoparticles	Large surface area, biocompatibility, specificity, stability, reproducibility, favorable selectivity, wide dynamic range and an ultralow detection limit	Label-free	CEACEA)	0.0003 ng mL^−1^	0.001–200 ng mL^−1^	[67]
GCE	AuNPs	AuNPs/ion liquid/hollowed TiO_2_ nano-shell	Specificity, repeatability, stability, storage, reproducibility, recovery for real sample test, ultralow detection limit, wide linear range, reliability, low-cost and on-site monitoring	Enzymatic	Cholesterol	6.30 × 10^−9^ M	8.33 × 10^−9^–4.17 × 10^−7^ M	[68]
GCE	AuNPs	NPCo/Co_3_O_4_–Au	Stability, reproducibility and immobilization of large amount of biomolecules	Label-free	DON	1 pg mL^−1^	5 pg mL^−1^–100 ng mL^−1^	[15]
GCE	AuNPs	MWCNTs-GO-Thi-Au	Enhanced ECL signal, high sensitivity, good selectivity and stability	Enzymatic	Cholesterol	50 nM	0.15–828 µM	[73]
GCE	AuNPs	KNbO_3_-AuNPs@Bi_2_S_3_	Strong ECL signal, sensitivity, stability, long-term stability, acceptable selectivity, precision and accuracy	Label-free	PSA	3 pg mL^−1^	0.0055 ng mL^−1^	[70]
GCE	AuNPs	GO/MWCNTs-COOH/Au@CeO_2_	High sensitivity, repeatability, selectivity, long-term stability, wide linear range	Label-free	CEA	0.02 ng mL^−1^	0.05–100 ng mL^−1^	[74]
GCE	AuNPs	AuNPs-CdSe QDs	Sensitivity, large surface area, rapid, acceptable precision, good stability, bioactivity and low-cost	Competitive	Phenylethanolamine A (PA)	0.0047 ng mL^−1^	0.02 –50 ng mL^−1^	[69]
GCE	AuNP	rGO/MWCNTs/AuNPs	Enhanced ECL signal intensity, high sensitivity, good reproducibility, stability and repeatability	Direct	Dopamine (DA)	0.067 µM	0.20–70 µM	[75]
GCE	AuNPs	AuNPs-CdSe QDs	Rapid and ultrasensitive, good stability, specificity and fabrication reproducibility	Competitive	Clenbuterol (CLB)	0.0084 ng mL^−1^	0.02–50 ng mL^−1^	[76]
GCE	AuNPs	Fe_3_O_4_ (Au-FrGO)	Large ECL signal, surface area, biocompatibility, sensitive response, excellent stability, repeatability and selectivity	Label-free	CEA	3.28 fg mL^−1^	0.01 pg mL^−1^–10 ng mL^−1^	[9]
GCE	AuNPs	petal-like CdS/AuNPs	high sensitivity, stability, good selectivity and wide linear range	Label-free	Immunoglobulin E	8.0 × 10^−14^ M	5.0 × 10^−13^–1.0 × 10^−9^ M	[77]
ITO	Gold nanodots	ABEI/GNDs/chitosan/COOH-MWCNTs	Strong and stable ECL signal, selectivity, stable and reliable response, extremely high sensitivity, satisfactory recovery	Label-free	N-Terminal Pro-brain natriuretic peptide (NT-proBNP)	3.86 fg mL^−1^	0.01–100 pg mL^−1^	[71]
GCE	AgNPs	luminol-AgNPs@OMC	Fast, sensitive, specific, stable and reliable	Label-free	Aflatoxin B1 (AFB1)	50 fg mL^−1^	0.1 pg mL^−1^–50 ng mL^−1^	[62]
GCE	Au@Ag nanorods	Au and Ag Bimetallic	Catalytic, sensitive, stable, specific and reproducible	Label-free	CEA	30 fg mL^−1^	0.1 pg mL^−1^–380 ng mL^−1^	[78]
GCE	nanoFe_3_O_4_	nanoFe_3_O_4_@GO	Good conductivity, magnetism, stability, reproducibility and regeneration	Label-free	Carbohydrate antigen 19-9 (CA19-9)	0.0005 U mL^−1^	0.001–5 U mL^−1^	[79]
GCE	Fe_3_O_4_	Fe_3_O_4_ (Au-FrGO)	Large ECL signal, specific surface area, biocompatibility, sensitive response, excellent stability, repeatability and selectivity	Label-free	CEA	3.28 fg mL^−1^	0.01 pg mL^−1^–10 ng mL^−1^	[9]
GCE	Fe_3_O_4_	(RGO)/Fe_3_O_4_/CdSe	Sensitivity, excellent reproducibility and stability, wide linear range and high selectivity	Label-free	Interleukin-6 (IL-6)	0.65 pg mL^−1^	0.002–20 ng mL^−1^	[10]
GCE	Fe_3_O_4_	CdS-Fe_3_O_4_	Sensitive response, wide linear range, low detection limit, rapid, specific, stable, reliable and ultrasensitive ECL	Label-free	Ochratoxin A (OTA)	2 pg mL^−1^	0.01–100 ng mL^−1^	[80]
GCE	Magnetic nanofibers/CNHs	Magnetic nanofibers-Fe_3_O_4_	Excellent electrical conductivity, large surface areas, variable porosity, amplified ECL, acceptable precision, reproducibility, biocompatibility, sensitivity, low detection limit and stability	Label-free	AFB1	0.02 ng mL^−1^	0.05–200 ng mL^−1^	[81]
Modified titanium ribbon	AuNCs	Au NCs/GR	High sensitivity, low detection limit, wide dynamic range, low toxicity, good regeneration and can be used with real samples	Direct	Pentachlorophenol (PCP)	0.1 fM	0.1 fM–0.1 nM	[82]
GCE	AuNCs	lum-AuNCs	100-folds enhancement of ECL, high specificity, good stability and potential clinical application	Direct	Alkaline phosphatase (ALP)	0.1 nM	0.3–12 nM	[83]

**Table 3:** Quantum dots-based ECL nanobiosensors.

Electrode	NMs/NSMs	Nanocomposites	Function	Assay Type	Bioanalyte	LOD	Range	Ref
GCE	CdS QDs	CdS-Fe_3_O_4_	Ultrasensitive ECL detection, wide linear range, low detection limit, rapid, specific, stable and reliable	Label-free	OTA	2 pg mL^−1^	0.01–100 ng mL^−1^	[80]
GCE	CdSe QDs	CdSe QDs/PICA-MWNT	Good biocompatibility, high ECL intensity, synergistic improvement of sensitivity, good selectivity and reproducibility	Label-free	Alpha-fetoprotein (AFP)	0.4 pg mL^−1^	0.002–2000 ng mL^−1^	[86]
GCE	CdSe QDs	HCNTs-Polyallylamine hydrochloride (PAH)-CdSe QDs	Signal amplification, increased surface area	Direct	DA	0.2 × 10^−9^ M	1.0 × 10^−9^–2.0 × 10^−5^ M	[87]
GCE	CdSe QDs	AuNPs-CdSe QDs	Electron transport accelerators, rapid, acceptable, precision, good stability, bioactivity, low-cost and lower detection limit	Competitive	PA	0.0047 ng mL^−1^	0.02–50 ng mL^−1^	[69]
GCE	CdSe QDs	AuNPs-CdSe QDs	Rapid, ultrasensitive, stability, specificity, fabrication, reproducibility and sensitivity	Competitive	CLB	0.0084 ng mL^−1^	0.02–50 ng mL^−1^	[76]
GCE	CdSe QDs	(RGO)/Fe_3_O_4_/CdSe	Sensitivity, stability, wide linear range, reproducibility and selectivity	Label-free	IL-6	0.65 pg mL^−1^	0.002–20 ng mL^−1^	[10]
GCE	CdTe QDs	Graphene nanosheets (GNs)/CdTe QDs	Sensitivity, selectivity, reproducibility and ideal stability	Enzymatic	organophosphate pesticides (OPs)	0.06 ng mL^−1^	0.2–10 ng mL^−1^	[88]
Au/AgrGO	Graphene QDs	Graphene QDs	Ultrasensitive ECL, excellent electron conductivity, stability, sensitivity and repeatability	Label-free	PSA	0.29 pg mL^−1^	1 pg mL^−1^–10 ng mL^−1^	[38]

**Table 4:** CNMs-, CNSMs- and NCs-based nanobiosensors.

Electrode	NMs/NSMs	NCs	Function	Assay type	Bioanalyte	LOD	Range	Ref
GCE	MWCNTs	MWCNTs-Cys-AuNPs	Signal amplification, reproducibility, stability, sensitivity and cost-effective	Direct	DPH	6.7 × 10^−9^ M	2 × 10^−8^–7.5 × 10^−4^ M	[72]
GCE	MWCNTs	MWCNT-Pt-luminol	ECL signal, sensitivity, fast analysis, stability and specificity	Label-free	CA19-9	0.00004 U mL^−1^	0.0001–10.0 U mL^−1^	[63]
GCW	MWCNTs	MWCNTs-Au	Signal amplification, selectivity, stability and low-cost	Direct	Bisphenol A	0.083 μM	0.25–100 μM	[61]
GCE	HCNTs	HCNTs-Polyallylamine hydrochloride (PAH)-CdSe QDs	Signal amplification, increased surface area, stability, sensitivity and excellent specificity	Direct	DA	0.2 × 10^−9^ M	1.0 × 10^−9^–2.0 × 10^−5^ M	[87]
GCE	Magnetic nanofibers/CNHs	CNHs	Excellent electrical conductivity, biocompatibility, large surface areas, variable porosity, amplified ECL, large antibody, acceptable precision, low detection limit, reproducibility and stability	Label-free	AFB1	0.02 ng mL^−1^	0.05–200 ng mL^−1^	[81]
GCE	MWCNTs	MWCNT-Pt-luminol	ECL signal, fast analysis, stability, reproducibility and specificity	Label-free	CA19-9	0.00004 U mL^−1^	0.0001–10.0 U mL^−1^	[85]
GCE	MWCNTs	nanoCo-MWCNTs	Sensitivity, economical and practical applications	Direct	Glucose	50 nM	0.5–600 μM	[86]
GCE	MWCNTs	MWCNTs-GO-Thi-Au	Enhancing ECL, high sensitivity and good selectivity	Enzymatic	Cholesterol	50 nM	0.15–828 µM	[73]
GCE	MWCNTs	GO/MWCNTs-COOH/Au@CeO_2_	High sensitivity, repeatability, accelerated electrons transfer, long-term stability and wide linear range	Label-free	CEA	0.02 ng mL^−1^	0.05–100 ng mL^−1^	[74]
ITO	MWCNTs	ABEI/GNDs/chitosan/COOH-MWCNTs	Strong and stable ECL signal, selectivity, stability and extremely high sensitivity	Label-free	NT-proBNP	3.86 fg mL^−1^	0.01–100 pg mL^−1^	[71]
GCE	MWCNTs	rGO/MWCNTs/AuNPs	Enhanced ECL signal intensity, high sensitivity, good reproducibility and repeatability	Direct	DA	0.067 µM	0.20–70 µM	[75]
GCE	GO	MWCNTs-GO-Thi-Au	Enhanced ECL, high sensitivity and excellent stability	Enzymatic	Cholesterol	50 nM	0.15–828 µM	[73]
GCE	GO	GO/MWCNTs-COOH/Au@CeO_2_	High sensitivity, accelerated electron transfer, selectivity and wide linear range	Label-free	CEA	0.02 ng mL^−1^	0.05–100 ng mL^−1^	[74]
GCE	GO	nanoFe_3_O_4_@GO	Good conductivity, magnetism, reproducibility and regeneration	Label-free	CA19-9	0.0005 U mL^−1^	0.001–5 U mL^−1^	[79]
GCE	rGO	rGO/MWCNTs/AuNPs	Enhanced ECL signal intensity, high sensitivity and repeatability	Direct	DA	0.067 µM	0.20–70 µM	[75]
GCE	rGO	Fe_3_O_4_ (Au-FrGO)	Increased ECL, specific surface area, biocompatibility, sensitive response, excellent stability and selectivity	Label-free	CEA	3.28 fg mL^−1^	0.01 pg mL^−1^–10 ng mL^−1^	[9]
GCE	rGO	(RGO)/Fe_3_O_4_/CdSe	Sensitivity, stability, excellent reproducibility and selectivity	Label-free	IL-6	0.65 pg mL^−1^	0.002–20 ng mL^−1^	[10]
GCE	GO	PtNFs/GO/GODx	High electrocatalytic activity, enhanced luminol ECL, reliability, high surface area, linear range and low detection limit	Direct	Glucose	2.8 μM	5–80 μM	[94]
QDs-SPE	CNOs	AuNPs@CNOs	High ECL, increased effective surface area for capturing and binding of antibodies, improved electron transmission rate, enhanced photon capture and highly sensitive	Label-free	β2M	1 fg mL^−1^	1 fg mL^−1^–100 ng mL^−1^	[7]
GCE	SWCNH	Aptamer/SWCNH	Quenching of ECL, sensitive, selective, simple, time-saving and cost-effective	Label-free	Adenosine triphosphate (ATP)	1 nM	5 nM–500 μM	[95]
GCE	OMCs	luminol-AgNPs@OMC	Fast, sensitive, specific, stable and reliable	Label-free	AFB1	50 fg mL^−1^	0.1 pg mL^−1^–50 ng mL^−1^	[62]
